# Abichromatic method for the enzymatic determination of serum triglycerides with the Gemsaec Fast analyser

**DOI:** 10.1155/S1463924680000600

**Published:** 1980

**Authors:** P. Bijster, H. L. Vader, C. L. J. Vink

**Affiliations:** Clinical Laboratory St. Joseph Hospital Aalsterweg 259 Eindhoven The Netherlands


dialyser membrane would continuously flex as samples of different protein concentrations are analysed – the peak height of a specimen does not necessarily reflect its creatinine level. In the authors' experience this produces many bizarre peak shapes within every batch and the last peak is always higher than expected. One way of minimising conformational changes in the dialyser membrane is to raise the waste donor stream of the dialyser. However, under these conditions small changes in conformation still occur due to the elasticity in the membrane, and the last peak in every batch is still higher than expected. A satisfactory solution to this problem has been found by minimising pressure differences between samples. A plasma creatinine analyser has been operated for the last 18 months using a sample diluent of sodium chloride (300 mmol/l) and 10 g/l of Brij 35. Over 75,000 specimens were analysed during this time. A higher concentration of 30 g/l is not used, in order to reduce the possibility of phase separation when samples of high total protein are analysed [1].

ACKNOWLEDGEMENT
The authors wish to acknowledge the financial support from the Medical Research Council of New Zealand and constructive discussions with Dr C. M. André.

REFERENCES
[1] Walmsley, T. A., *Clin. Chim. Acta*, 1979, **99**, 53-57.
[2] Technicon Method No. AAII-11, 1970, Technicon Instrument Corporation, Tarrytown, New York.

### A bichromatic method for the enzymatic determination of serum triglycerides with the Gemsaec Fast analyser

**P. Bijster*, H. L. Vader and C. L. J. Vink**
*Clinical Laboratory, St. Joseph Hospital, Aalsterweg 259, Eindhoven (The Netherlands).*

### Introduction

Fully enzymatic methods for the determination of serum triglycerides may be carried out in either a kinetic or an equilibrium mode. Both kinetic and equilibrium methods have been adapted for use on centrifugal analysers. Kinetic (or fixed-time rate) analyses are carried out by adapting the reaction conditions to achieve first or pseudo-first-order kinetics [1-5]. Equilibrium analyses on centrifgual analysers deal with some special problems concerning the initial absorbance reading [1,6]. If a slight lag-phase is present in the first stage of the reaction some analysers can read an initial absorbance during this time. Otherwise 'zero-time' extrapolation procedures must be used despite the difficulties these present. Another drawback of equilibrium analyses, especially for centrifugal analysers, is the long reaction time.

A fully enzymatic equilibrium method adapted to the Gemsaec Fast analyser which takes less than two minutes of instrument time is described. It is a modification of the method described by Bucolo *et al* [7]. In this procedure the triglycerides are released and hydrolysed by a mixture of lipase and α-chymotrypsin. Subsequently, the released glycerol is converted to glycerol-1-phosphate in the presence of glycerol kinase and ATP. Phosphoenol pyruvate, included in the reagent, is converted by pyruvatekinase to pyruvate resulting in the regeneration ATP from ADP. The reduction of pyruvate to lactate in the presence of lactate dehydrogenase with concurrent oxidation of $NADH_2$ to $NAD^+$ is used as the indicator reaction. Both the hydrolysis and the elimination of glycerol are carried out at 37°C in the transfer disc outside the analyser within 10 minutes.

To minimise optical interferences, the absorbance of the reaction mixture is read after 10 minutes at two wavelengths, namely 340 nm and 380 nm. There is no optical interference by lipemic sera as the absorbances are determined after complete hydrolysis of the lipids. The concentration of triglycerides is calculated by subtracting the absorbance differences (A 340 nm – A 380 nm) of the reagent blank and determinations compared to that of a glycerol standard.

### Materials

### Eskalab triglyceride reagent

Eskalab substrate vials (89804) and Eskalab glycerol kinase vials (89802), obtained from Smith Kline Instruments, Inc., Sunnyvale, California 94086, USA, were dissolved in bi-distilled water according to the manufacturer's recommendations. The reactants in the reagent mixture have the following approximate concentrations:

| | |
|---|---|
| *Substrate mixture* | |
| Reduced nicotinamide adenine dinucleotide | 0.3 mmol/l |
| Pyruvate kinase | 0.8 x $10^3$ IU |
| Lactate dehydrogenase | 0.7 x $10^3$ IU |
| $Mg^{2+}$ | 6 mmol/l |
| Lipase | 300 x $10^3$ IU |
| α-chymotrypsin | 13 x $10^3$ IU |
| Phosphoenol pyruvate | 0.8 mmol/l |
| Bovine serum albumin | 1.7 g/l |
| Adenosine triphosphate | 0.5 mmol/l |
| Buffer: (pH 7.1 ± 0.2) | |
| Potassium dihydrogen phosphate | 10 mmol/l |
| di-Potassium hydrogen phosphate | 63 mmol/l |

*Glycerol kinase suspension*
Glycerol kinase not less than 20 IU/vial.

**Correspondence to this author*

*Combined reagent*
10μl Of the glycerol kinase suspension were added per ml of the substrate solution and the reagent was mixed.

**Glycerol standards**
100% Glycerol, purchased from Fluka A. G., Buchs S. G. (Prod. no 49770) was used as the primary standard. The water content of the 100% glycerol was checked by measuring the relative viscosity at 20.0°C [8]. As a secondary standard Precimat glycerol standard, obtained from Boehringer Mannheim GmbH, Germany, was used. This is a stabilised aqueous solution of glycerol which contains 2.29 mmol/l glycerol, corresponding to a triglyceride concentration of 2.29 mmol/l. This standard was checked with aqueous dilutions (v/v) of 100% glycerol.

**Ethanolic potassium hydroxide (ca. 0.5 mol/l)**
3.30 g 85% Potassium hydroxide p.a. (Merck art. 5033) were dissolved in 10 ml bidistilled water, under cooling with tap water. 96% Ethanol p.a. (Merck art. 972) was added to a final volume of 100 ml.

**Magnesium sulphate solution (0.15 mol/l)**
3.70 g Magnesium sulphate 7 aq. p.a. (Merck art. 5886) were dissolved in 50 ml bidistilled water and bidistilled water was added to a final volume of 100 ml.

**Boehringer test-combination triglycerides**
All reagents in this kit (no. 124966), purchased from Boehringer Mannheim GmbH, Germany, were dissolved in bidistilled water according to the manufacturer's recommendations.

**Turbidity standard**
5 g Hexamethylene tetramine (Merck art. 4343) were dissolved in 30 ml bidistilled water and brought to a final volume of 50 ml. 0.5 g Hydrazine sulphate (BDH 'analar' no. 10121) were dissolved in the same way to a final volume of 50 ml. Both solutions were combined (1:1) to a stock-standard. The working-standard was prepared by dilution of 12 ml stock-standard with bidistilled water to a volume of 50 ml.

### Apparatus

1. ENI-Gemsaec centrifugal analyser obtained from Electro-Nucleonics Inc., Fairfield, New Jersey 07006, USA, with optional addition of a Digital Decwriter II and an ENI-Gemsaec rotoloader IV with Micromedic Systems automatic pipettes.
2. LKB ultrolab system 2074 calculating absorptiometer obtained from LKB Produkter AB, Bromma 1, Sweden.
3. A Vitatron Photometer UFD, was used as a nephelometer without optical filters, obtained from Vitatron, Dieren, The Netherlands.

### Methods

The transfer discs are prewarmed to the incubation temperature (37°C) before they are loaded. 10 μl Bidistilled water, standard, control or serum sample, dispensed with 80 μl saline followed by 500 μl combined reagent (see Materials) are pipetted into well C of the transfer disc. Positions 1, 2 and 3 of the transfer disc contain bidistilled water, reagent blank and a glycerol standard, respectively.

After an incubation at 37°C for 10 minutes the hydrolysis of the triglycerides and elimination of glycerol has been completed and the transfer disc is placed into the Gemsaec analyser and the run is started. The analyser is operated at 37°C and the first reading at 340 nm is taken after 10 seconds (IR = 10). Thirty seconds after the vacuum blast (mixing) the wavelength is changed to 380 nm and the second reading is taken 60 seconds after the first reading (RI = 60, NR = 2).

The computer instructions are as follows:

| | | | | | |
|---|---|---|---|---|---|
| IR | = | 10 | AD | = | 3 |
| RI | = | 60 | CD | = | 2 |
| NR | = | 2 | HI | = | 2.5 |
| SC | = | 2.29 | LO | = | 0.5 |
| KT | = | 1.0 | SA | = | 0.4 |
| TF | = | 1.0 | RM | = | 2 |
| TC | = | 1 | XX | = | 1 |

To calculate correctly the total glycerol concentration of the samples, the first printout must be disregarded and an additional computer program must be used (see Manual SKI). This program performs the following calculations using the absorbances stored in the computer memory:

1. $A_{340} - A_{380}$ = Delta in which $A_{340}$ = absorbance at 340nm; $A_{380}$ = absorbance at 380nm
2. $\text{Delta}_{\text{blank}} - \text{Delta}_{\text{samples}} = A_{\text{corrected}}$
3. $\frac{\text{A corrected unknown}}{\text{A corrected standard}}$ x glycerol concentration of standard (mmol/l) = total glycerol concentration of unknown (mmol/l)

Results were compared to the manual enzymatic method after saponification with ethanolic KOH (see Materials) which was performed according to the manufacturers recommendations. In routine clinical chemistry, the triglyceride concentration is obtained by subtracting 0.11 mmol/l as correction for the mean free glycerol concentration, from the measured value [9]. Otherwise the concentration of 'free' glycerol may be estimated separately.

### Experiments and results

**Wavelength choice**
The glycerol concentration of an aqueous glycerol solution is generally measured by determining the absorbance difference between the reagent blank and the sample relative to the absorbance difference between the reagent blank and a standard at one wavelength (340 nm). When optical 'normal' sera diluted in the combined reagent as described earlier are analysed in this way, the triglyceride concentration will be decreased by approximately 0.1 – 0.2 mmol/l.

As it is impossible to read the initial absorbance of the reaction mixture at the Gemsaec Fast analyser, an alternative approach is to measure the absorbance of the reaction mixture at two wavelengths, after completion of hydrolysis and elimination of glycerol. The choice of the second wavelength, next to the fixed 340 nm, is based on several factors. Firstly, interference caused by haemolytic and icteric samples must be minimised. Secondly, the sensitivity of the assay must be guaranteed and thirdly, the practical handling of the assay on the Gemsaec analyser is of importance.

To evaluate the possible colorimetric interferences spectra were measured of haemolytic and icteric sera in the reaction mixture without glycerolkinase against the same mixture without these pigments. These experiments showed that minimum interference of haemolysis was given if the second wavelength is about 375 nm. To evaluate the interference caused by icteric samples spectra of icteric sera with a high and low percentage conjugated bilirubin were measured. As the absorption minimum for both spectra is about 340 nm it appeared that for minimising the interference of bilirubin the best choice for the second wavelength is about 340 nm.

The sensitivity of the assay is dependent on the absorbance difference between 340 nm and the second wavelength. The choice of a high wavelength (>390 nm) will guarantee good sensitivity (a large absorbance difference) but, as stated above, will cause erroneous results especially when assaying icteric samples. The choice of a low wavelength (<370 nm) will result in minimal interference caused by icteric samples but pronounced interference caused by haemolytic samples and loss of sensitivity. In practice, the choice of 380 nm as the second wavelength offers a reasonable compromise. The interference of pigments at this wavelength is acceptable and the sensitivity is guaranteed as the corrected absorbance (Delta blank – Delta sample) for a triglyceride concentration of 2.3 mmol/l (reference range 0.84 – 1.94) is 36% higher than the corrected absorbance at 370 nm. Furthermore, changing the wavelength during the assay on the Gemsaec spectrophotometer from 340 nm to 380 nm and calibration and checking the cuvettes at both wavelengths presents no practical problems as the same filter range can be used.

The repeated adjustment of the wavelength scale at the chosen wavelengths is sufficiently exact.

### Linearity

A series of glycerol standards was prepared by diluting 100% glycerol (primary standard) with bidistilled water (v/v). The primary standards were assayed four times and the glycerol concentrations were calculated against the secondary Precimat glycerol standard. The results (Figure 1) show that there is good linearity over the range of glycerol concentrations from 0 up to 7.5 mmol/l (max. deviation from calculated glycerol concentration 0.5%). Furthermore, the results show that the stated glycerol concentration of the Precimat standard is correct.

### Precision

The within-run precision was determined by assaying serum pools with different triglyceride concentrations in three runs. In each run aliquots of the same serum were pipetted in 13 wells of the transfer disc. The results are presented in Table 1. The day-to-day precision was determined by assaying the control serum liponorm (Boehringer Mannheim GmbH) once a day in duplicate. The results over a period of 32 days are also shown in Table 1.

### Accuracy

The automated bichromatic enzymatic method was compared to a manual enzymatic method, the Boehringer test-combination triglycerides. Seventy-three sera were analysed by both methods. The results are shown in Figure 2. The correlation curve, calculated according to Lindley [10] assuming that the variances in x and y are equal, is described by the equation

$$y = 1.03x - 0.05$$

with a coefficient of correlation of 0.996. The data followed a normal distribution, checked with the Kolmogorov-Smirnov-test [11] ($\alpha = 0.05$).

### Interference of haemoglobin

Any interference of haemolysis with this bichromatic method was investigated. Blood from a healthy donor was centrifuged after clotting and the blood clot was washed twice with 0.9 g/l NaCl. Erythrocytes were haemolysed by

**Table 1. Within-run and day-to-day precision of three sera and one control serum, respectively**

| | Within-run | | | Day-to-day |
|---|---|---|---|---|
| Sample | A | B | C | Liponorm |
| Mean (mmol/l) concentration of triglycerides | 0.49 | 1.81 | 3.19 | 1.93 |
| Standard deviation (mmol/l) | 0.012 | 0.026 | 0.048 | 0.048 |
| Coefficient of variation (%) | 2.6 | 1.4 | 1.5 | 2.5 |
| Number of samples | 13 | 13 | 13 | – |
| Number of days | – | – | – | 32 |

**Table 2. Influence of haemolysis on the determination of triglycerides**

| Haemoglobin concentration (mmol/l) | Qualitative haemolysis according to Frank *et al* [12] | Expected triglyceride concentration (mmol/l) | Measured triglyceride concentration (mmol/l) | Apparent increase in triglyceride concentration (mmol/l) |
|---|---|---|---|---|
| 0.00 | – | 2.16 | 2.16 | 0.00 |
| 0.03 | + | 2.15 | 2.18 | 0.03 |
| 0.06 | 2+ | 2.14 | 2.20 | 0.06 |
| 0.12 | 3+ | 2.12 | 2.21 | 0.09 |
| 0.23 | 4+ | 2.08 | 2.23 | 0.15 |

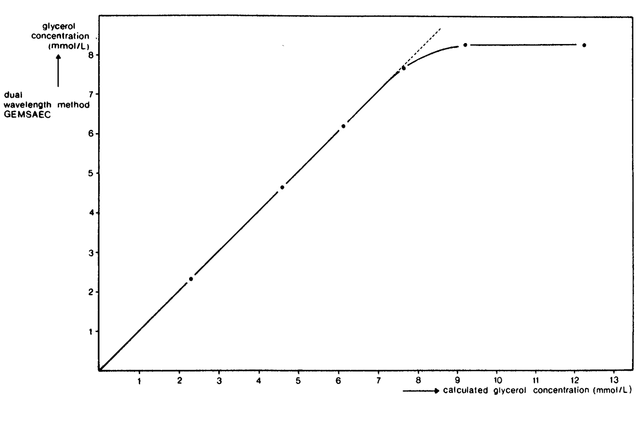

*Figure 1. Glycerol concentrations measured by the dual wavelength method on the Gemsaec Fast analyser of a series of glycerol standards prepared by diluting 100% glycerol with bidistilled water (v/v).*

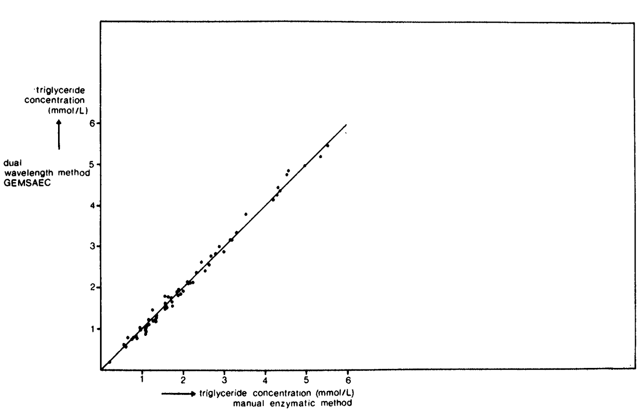

*Figure 2. Comparison of the triglyceride concentrations of 73 sera analysed with the dual wavelength method on the Gemsaec Fast analyser and with a manual enzymatic method after saponification with ethanolic KOH.*

adding bidistilled water and the suspension was homogenised in a mortar. After centrifuging the suspension five times for 30 minutes at 4000 rpm the haemoglobin solution had a haemoglobin concentration of 5.5 mmol/1 and a triglyceride concentration of 0.1 mmol/1. A series of dilutions with pooled serum was prepared. The expected triglyceride concentration of the haemolytic sera were calculated from the triglyceride concentration of the pooled serum (2.16 mmol/1) and the dilution factors obtained after the addition of the haemoglobin solutions. The triglyceride concentrations of the different haemolytic serum samples were determined in duplicate on the Gemsaec analyser by the bichromatic enzymatic method. The four haemolytic samples were categorised qualitatively in four classes (1+, 2+, 3+ and 4+) according to Frank *et al* [12]. Table 2 shows the results of these experiments. From these results it is evident that the presence of haemoglobin in serum results in an apparent increase of the triglyceride concentration. The authors consider that the influence of haemolysis which is acceptable for practical purposes as the triglyceride concentration of a very haemolytic (4+) sample (which rarely occurs) with an expected triglyceride concentration of 2.00 mmol/1 (reference range 0.84 – 1.94 mmol/1) will be increased by about 8%.

### Interference of bilirubin

In order to investigate the interference with the bichromatic method caused by bilirubin, a series of dilutions with saline were made of four different icteric sera, with a high percentage conjugated bilirubin (mean 73%). The diluted samples were assayed on the Gemsaec analyser in duplicate with substrate without the addition of glycerol kinase to evaluate the optical interference. The results are presented in Table 3. From these results it is evident that the presence of bilirubin in serum results in an apparent increase of triglyceride concentration. For example, the triglyceride concentration of an icteric serum with a total bilirubin concentration of 100 μmol/l (reference range 5–14 μmol/l) and an expected triglyceride concentration of 2.00 mmol/l (reference range 0.84 – 1.94 mmol/l) will be increased by about 10% when assayed with this method. As the spectra at about 340 nm of sera with high concentrations of unconjugated bilirubin in the reaction mixture are almost the same as the spectra of sera with high concentrations of conjugated bilirubin, the interference of unconjugated bilirubin on the assay is not quantified.

### Interference by turbidity

A series of turbid samples prepared by dilution of a strongly lipemic serum pool of 10 patients (triglyceride concentration 10.1 mmol/l) with saline was assayed, to evaluate the capability of the combined reagent to clear up sera with a high content of chylomicrons. The turbidity of the samples was measured in duplicate on a nephelometer, that was adjusted to an arbitrary transmission of 40% with the turbidity standard. The samples were analysed in duplicate on a Gemsaec analyser diluted with combined reagent without the addition of glycerol kinase to evaluate the influence of turbidity on the corrected absorbance. The results are shown in Table 4. From the results it is clear that even strongly lipemic sera are sufficiently cleared by the combined reagent. For example, the triglyceride concentration of the 2 x diluted pooled serum (strongly lipemic) with an expected triglyceride concentration of 5.0 mmol/1 was decreased by about 1.5%. Only extreme milky sera cause falsely lowered triglyceride concentrations but this will not cause erroneous results as these sera will have a high triglyceride concentration and dilution will be necessary to prevent crossing the upper limit of linearity (7.5 mmol/1).

**Table 3. Influence of total bilirubin concentration on the determination of triglycerides**

| Total bilirubin concentration (μmol/1) | A corrected ± SD (Delta blank – Delta sample) | Apparent increase in triglyceride concentration (mmol/1) |
|---|---|---|
| 0 | 0.000 ± 0.001 | 0.00 |
| 50 | 0.007 ± 0.001 | 0.08 |
| 100 | 0.016 ± 0.001 | 0.19 |
| 150 | 0.026 ± 0.002 | 0.32 |
| 200 | 0.036 ± 0.003 | 0.44 |
| 250 | 0.047 ± 0.005 | 0.57 |

**Table 4. Influence of turbidity on the determination of triglycerides**

| Dilution factor lipemic serum pool | Nephelometry % transmission | A corrected (Delta blank –Delta sample) | Apparent decrease in triglyceride concentration (mmol/1) |
|---|---|---|---|
| undiluted | 21.7 | -0.017 | 0.21 |
| 2x | 29.3 | -0.007 | 0.08 |
| 4x | 29.6 | -0.003 | 0.04 |
| 8x | 23.2 | -0.001 | 0.01 |
| 12x | 17.0 | 0.000 | 0.00 |
| 18x | 12.1 | 0.000 | 0.00 |

### Discussion

An enzymatic analysis based on an equilibrium measurement has the advantage over a kinetic measurement that temperature or drugs have no influence on the kinetics of the reaction. In addition, a linear reaction curve is not necessary. The difficulty in adapting an equilibrium analysis to the Gemsaec analyser is that it is impossible to take an early absorbance reading. Chong-Kit *et al* [6] have solved this problem and introduced a 'zero-time extrapolation' by means of an extrapolation factor. The authors evaluated a bichromatic reading after completion of the reactions which replaces the 'zero-time extrapolation'. A bichromatic reading may introduce errors caused by pigments and/or turbidity. Data presented here shows that these interferences caused by haemoglobin and bilirubin are not completely minimised by this bichromatic reading. However, the influence of haemolysis only becomes important at haemoglobin concentrations that seldom occur in practice and the authors consider that this method is acceptable for practical purposes. The interference of bilirubin is the result of the choice of the second wavelength. There is an almost linear relation between the bilirubin concentration and the apparent increase in triglyceride concentration; for every 50 μmol/l bilirubin the triglyceride concentration of an icteric sample is increased by 0.1 mmol/1. The interference caused by lipemic turbidity is excluded as the absorbances are determined after complete hydrolysis of the lipids.

The disadvantage of a long hydrolysis time (10 minutes) is limited because the incubation takes place outside the Gemsaec analyser. During this incubation the preceding batch is run, calculated and the necessary administration can be performed. In principle this enzymatic analysis of serum triglycerides can be performed on every spectrophotometer with variable wavelength setting and on the recently introduced bichromatic analysers after hydrolysis of glycerides. The choice for the Gemsaec analyser is based on the advantage this system offers (economic reagent use, good precision, easy calculation of results, clear presentation, statistical evaluation).

ACKNOWLEDGEMENTS
The authors wish to thank Gist-Brocades Farmaca Nederland BV and Smith Kline Instruments, Inc. for their cooperation in this study and Mrs Geboers-van der Have for her assistance in the preparation of the manuscript.

REFERENCES
[1] Tiffany, T. O., Morton, J. M., Hall, E. M. and Garrett, A. S., *Clinical Chemistry*, 1974, **20**, 476.
[2] Ziegenhorn, J., *Clinical Chemistry*, 1975, **21**, 1627.
[3] Wentz, P. W., Cross, R. E. and Savory, J., *Clinical Chemistry*, 1976, **22**, 188.
[4] Grossman, S. H., Mollo, E. and Ertingshausen, G., *Clinical Chemistry*, 1976, **22**, 1310.
[5] Wakayama, J. E. and Swanson, J. R. *Clinical Chemistry*, 1977, **23**, 223.
[6] Chong-Kit, R. and McLaughlin, P., *Clinical Chemistry*, 1974, **20**, 1454.
[7] Bucolo, G. and David, H., *Clinical Chemistry*, 1973, **19**, 476.
[8] Wolf, A. V., Brown, M. G. and Prentiss, P. G. in 'Handbook of Chemistry and Physics' Ed. Weast, R. C., CRC Press, Cleveland, Ohio, D-206.
[9] Stinshoff, K., Weisshaar, D., Staehler, F., Hesse, D., Gruber, W. and Steiner, E., *Clinical Chemistry*, 1977, **23**, 1029.
[10] Lindley, D. V., *Supplement Journal Royal Statistical Society*, 1947, **9**, 218.
[11] Massey, F. J., *Journal American Statistical Association*, 1951, **46**, 68.
[12] Frank, J. J., Bermes, E. W., Bickel, M. J. and Watkins, B. F., *Clinical Chemistry*, 1978, **24**, 1966.

### A clinical appraisal of the Greiner G300 clinical chemistry analyser

**M.J. Hallworth and S.G. Archer**
*Department of Clinical Biochemistry, Addenbrooke's Hospital, Hills Road, Cambridge CB2 2QR.*

**and J.G. Lines**
*Department of Clinical Chemistry, William Harvey Hospital, Ashford, Kent TN24 0LZ.*

### Introduction

The demands on clinical chemistry laboratories over the last two decades have grown so rapidly that automated multiple analysis upon single samples is now the only practicable method of providing a routine clinical chemistry service in all but the smallest hospitals. A large variety of equipment is commercially available for performing this task rapidly and economically, whilst maintaining high standards of analytical quality. Any new instrument must therefore equal or better the established standards of precision, and must use analytical methods producing results as close as possible to reference methods for each analyte.

The work which follows sets out to determine the precision and accuracy of the Greiner G300 analyser in routine use and to examine both the analytical capability of the instrument and also the contribution it can make towards handling the workload of the clinical chemistry laboratory.

The Greiner G300 analyser is a third-generation instrument, which essentially comprises up-dated modifications of the Greiner Selective Analyser (GSA II) which has been available for six years. In the United Kingdom, the GSA II was first formally evaluated by Skinner and Wilding [I] in 1975, and a more comprehensive evaluation has recently been produced by Carlyle *et al* [2] who made their study over a considerable period of routine use.

The present evaluation was planned as a rapid assessment of the clinical utility of the new G300. Following exhibition at the 3rd European Congress of Clinical Chemistry (Brighton, June 1979), a prototype instrument was transported to Cambridge and was operational for this clinical trial the day after arrival. Over the next five days a series of experiments designed to rapidly assess the performance of the G300 under routine conditions was performed.

### Instrument description

The instrument is a discrete analytical system capable of performing up to 30 different analyses on a single specimen. Methods for 20 analyses are already fully documented, and the repertoire is being extended by further tests as they are adapted to the G300 from the GSA II.

The G300 is physically smaller than the GSA II and has improved process control facilities. The number of analyses on each specimen and the rate of analysis remain the same, but the handling of emergency and paediatric samples is faster and more convenient than on the GSA II. A washing cycle for process tubes is a new feature of the G300 and contrasts advantageously to the disposable process tubes used by the GSA II. Carlyle *et al* [2] found that the cost of process tubes considerably exceeded the cost of reagents for most tests, therefore the economic saving is appreciable.

The G300 comprises a 30 place sample carrier and sampling unit, a conveyor belt system on which the process tubes used for reactions are supported and moved, and a photometer unit with associated electronics. The analysis time from sampling to measurement of the final optical density is 13.4 minutes. The reaction time is variable between 36 seconds and 12.6 minutes according to the position of the reagent dispensers within the incubator unit, and the optical density may be read at the wavelength of any one of eight mercury lines, between 334 and 623 nm. The same sample can be dispensed into a pair of process tubes, and up to four different reagents added to each tube. In this manner blank measurements can be determined when required. The operator selects the tests to be performed on each specimen by means of a keyboard next to the sample tray. Commonly-requested groups of analyses (biochemical profiles) can be selected by means of a single key, and some examples of profiling rates are shown in Table 1. It is possible to introduce any urgent investigation into the middle of the batch of other analyses, or to perform only a few necessary tests on a small sample of serum, while performing a wider profile on the remainder of the batch. Emergency samples are placed in the next available space in the sample tray, the operator identifies the sample as an emergency, and keys in the test required: the instrument then positions the emergency samples under the sampling unit, samples sufficient serum

